# Transcriptome Analysis Reveals the Genes Related to Pollen Abortion in a Cytoplasmic Male-Sterile Soybean (*Glycine max* (L.) Merr.)

**DOI:** 10.3390/ijms232012227

**Published:** 2022-10-13

**Authors:** Zhiyuan Bai, Xianlong Ding, Ruijun Zhang, Yuhua Yang, Baoguo Wei, Shouping Yang, Junyi Gai

**Affiliations:** 1 Soybean Research Institute, National Center for Soybean Improvement, Key Laboratory of Biology and Genetic Improvement of Soybean (General, Ministry of Agriculture and Rural Affairs of the People’s Republic of China), State Key Laboratory of Crop Genetics and Germplasm Enhancement, Jiangsu Collaborative Innovation Center for Modern Crop Production, College of Agriculture, Nanjing Agricultural University, Nanjing 210095, China; 2 Center for Agricultural Genetic Resources Research, Shanxi Agricultural University, Taiyuan 030031, China

**Keywords:** soybean (*Glycine max* (L.) Merr.), cytoplasmic male sterility, pollen abortion, gene expression, RNA sequencing

## Abstract

Cytoplasmic male sterility (CMS) lays a foundation for the utilization of heterosis in soybean. The soybean CMS line SXCMS5A is an excellent CMS line exhibiting 100% male sterility. Cytological analysis revealed that in SXCMS5A compared to its maintainer SXCMS5B, its tapetum was vacuolated and abnormally developed. To identify the genes and metabolic pathways involving in pollen abortion of SXCMS5A, a comparative transcriptome analysis was conducted between SXCMS5A and SXCMS5B using flower buds. A total of 372,973,796 high quality clean reads were obtained from 6 samples (3 replicates for each material), and 840 differentially expressed genes (DEGs) were identified, including 658 downregulated and 182 upregulated ones in SXCMS5A compared to SXCMS5B. Among them, 13 DEGs, i.e., 12 open reading frames (ORFs) and 1 *COX2*, were mitochondrial genome genes in which *ORF178* and *ORF103c* were upregulated in CMS lines and had transmembrane domain(s), therefore, identified as CMS candidate mitochondrial genes of SXCMS5A. Furthermore, numerous DEGs were associated with pollen wall development, carbohydrate metabolism, sugar transport, reactive oxygen species (ROS) metabolism and transcription factor. Some of them were further confirmed by quantitative real time PCR analysis between CMS lines with the same cytoplasmic source as SXCMS5A and their respective maintainer lines. The amount of soluble sugar and adenosine triphosphate and the activity of catalase and ascorbic acid oxidase showed that energy supply and ROS scavenging decreased in SXCMS5A compared to SXCMS5B. These findings provide valuable information for further understanding the molecular mechanism regulating the pollen abortion of soybean CMS.

## 1. Introduction

A common biological phenomenon in nature, heterosis serves as an efficient agricultural approach for increasing crop yield. Utilization of heterosis in rice and corn could increase crop yield by 15% to 50% [1,2]. Research on utilization of soybean heterosis started late and progressed slowly. The discovery of soybean cytoplasmic male sterility (CMS) laid a foundation for the utilization of soybean heterosis [3,4]. At present, a number of research institutions in China had realized a three-line support system for hybrid soybean production [5,6].

Plant male sterility refers to the phenomenon that plants cannot produce functional pollen. Plant male sterility could be used not only as an important tool for heterosis utilization [7] but also as an ideal material for studying plant reproductive development [8,9]. Research indicated that male sterility was an extremely complex process, with diverse abortion forms and degrees [10,11]. Male sterility in plants was caused mainly by abnormal function of genes in nucleus or cytoplasm involving in pollen development, while toxic proteins, insufficient energy supply, abnormal programmed cell death (PCD), and other factors might lead to abnormal plant fertility [12,13,14,15,16]. In view of the complexity of male sterility in plants, it was very difficult to analyze the genetic mechanism from the perspective of individual genes by conventional methods. The transcriptome refers to the sum of all RNA transcribed by a specific cell under a certain functional state, and it thus can provide information on gene expression, gene regulation, and amino acid content [17]. The study of transcriptomics could screen and find the target genes regulating biological traits, infer the function of corresponding unknown genes, and reveal the action and molecular mechanism of genes in biological processes, which had been widely used in the study of plant male sterility [18,19,20]. However, there were still few reports on transcriptomics between soybean cytoplasmic male sterile lines and maintainer lines. Soybean cytoplasmic male sterile line NJCMS1A had been studied, but its sequencing library construction only involved nuclear genome [21]. The underlying molecular mechanism of CMS and the genes related to pollen abortion in soybean remains unclear.

The soybean CMS line SXCMS5A is a new male-sterile line successfully transferred from the variety JY20 with H3A cytoplasm. In the present study, we performed transcriptomic analyses of SXCMS5A vs. SXCMS5B, combined with quantitative real time PCR (qRT-PCR) analysis, cyto-morphological characteristic and enzyme activity assay, and substance content analysis in order to reveal the male sterility mechanism of SXCMS5A. We aimed to identify differences between CMS line SXCMS5A and its maintainer SXCMS5B at the transcriptional level and to find important differentially expressed genes (DEGs) and metabolic pathways related to pollen abortion. These findings might contribute to greater understanding of the molecular mechanism underlying CMS and provide useful information to facilitate progress in hybrid breeding in soybean.

## 2. Results

### 2.1. Comparison of the Cyto-Morphological Characteristics between Soybean CMS Line SXCMS5A and Its Maintainer SXCMS5B

**Figure 1:**
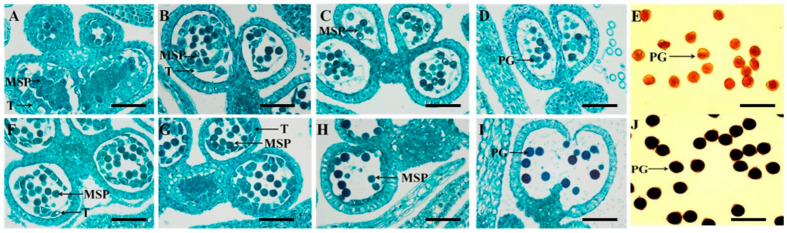
Microscopic observations of anthers from the soybean cytoplasmic male sterility (CMS) line SXCMS5A and its maintainer SXCMS5B. (**A**–**D**) Transverse sections of sterile anthers; abnormal tapetum and abnormal anthers developed in SXCMS5A. (**E**) Mature pollen grains stained by I_2_-KI in SXCMS5A. (**F**–**I**) Transverse sections of fertile anthers; normal tapetum and normal anthers developed in SXCMS5B. (**J**) Mature pollen grains stained by I_2_-KI in SXCMS5B. MSP, microspore; T, tapetum; PG, pollen grain; Bars = 20 μm.

In order to describe the cyto-morphological characteristics of pollen abortion of soybean CMS line SXCMS5A, the flower buds of SXCMS5A and SXCMS5B were observed and compared by paraffin sections. As shown in Figure 1A, at the tetrad stage, the tapetum cells of SXCMS5A were closely arranged, vacuolated, expanded inward, and tended to squeeze microspores. Subsequently, the tapetum was gradually broken and disintegrated, and clear contours and disintegrated fragments could be observed (Figure 1B,C). After that, the diaphragm between the two chambers did not open. And there were signs of vacuolization (Figure 1D). The pollen grains were abnormally developed and could not be stained by I_2_-KI (Figure 1E). In contrast, the tapetum of SXCMS5B normally initiated PCD (Figure 1F). Subsequently, the tapetum continued to degrade (Figure 1G). After that, microspores gradually developed and matured, the diaphragm between the two chambers opened normally (Figure 1H,I). The pollen grains developed normally and could be stained by I_2_-KI (Figure 1J). It was speculated that the tapetum of soybean CMS line SXCMS5A was vacuolated and abnormally developed, which could not provide necessary nutrients for microspore development, resulting in abnormal pollen development.

### 2.2. Transcriptome Sequencing, Sequence Alignment and Quality Evaluation

To further understand the molecular mechanism of CMS in soybean, RNA sequencing (RNA-Seq) analysis of flower buds of SXCMS5A and SXCMS5B was conducted using Illumina technology. As shown in Table S1, 58.97 Gb clean data and 393,126,454 clean reads were obtained from 6 samples. After strict filtering of the original data, 54.15 GB high quality clean data and 372,973,796 high quality clean reads were obtained. The average percentages of Q20 base, Q30 base, and GC content of all samples were 97.52%, 93.37% and 43.93%, respectively, indicating that the sequencing data was high quality to meet the standards for subsequent gene function analysis. Next, Tophat2 software was used to compare the filtered ribosomal reads to the reference genome. A total of 331,374,194 mapped reads were obtained, with an average matching rate of 89.98%. Pearson correlation coefficient analysis revealed that the correlation coefficients (R^2^) value between samples was greater than 0.97, showing that the expression mode of SXCMS5A was very close to SXCMS5B (Figure S1).

### 2.3. Identification and Confirmation of DEGs

**Figure 2:**
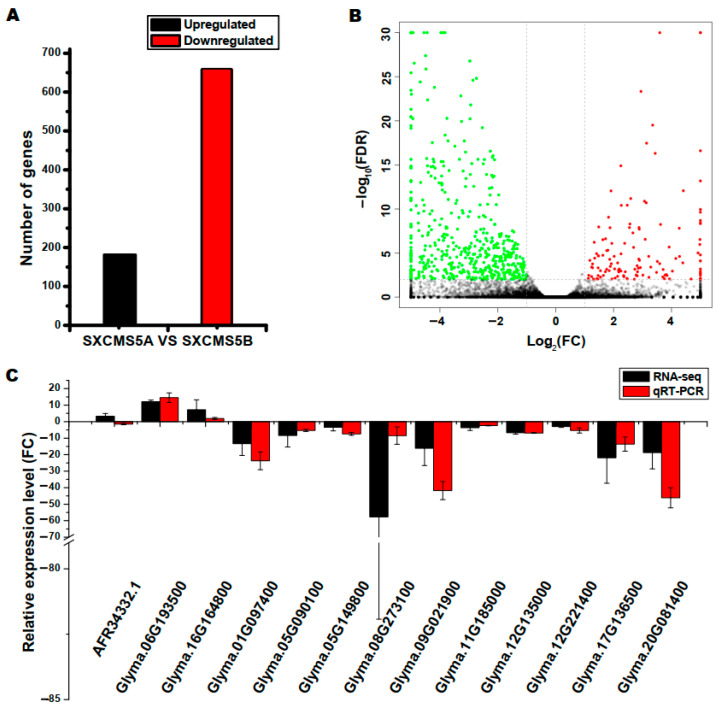
Analysis of differentially expressed genes (DEGs) between SXCMS5A and SXCMS5B. (**A**) Number of upregulated and downregulated DEGs. (**B**) Volcano plot comparing DEGs. Red dots, green dots, and black dots indicated DEGs that were significantly upregulated, significantly downregulated, or showed no significant difference in expression, respectively. (**C**) Relative expression level of selected DEGs. The *y*-axis indicated relative mRNA expression level, determined by RNA sequencing (RNA-seq) and quantitative real time PCR (qRT-PCR) analysis. The results were obtained from three biological replicates. FC, fold change; FDR, false discovery rate.

To identify putative DEGs between SXCMS5A and SXCMS5B, the thresholds of “False Discovery Rate (FDR) < 0.05 and |log_2_ Fold Change (FC)| > 1” was used to screen for DEGs. There were 840 DEGs between SXCMS5A and SXCMS5B, among which 658 downregulated and 182 upregulated in SXCMS5A compared to SXCMS5B (Figure 2A; Table S2). The expression of most DEGs in SXCMS5A was downregulated compared to SXCMS5B (Figure 2B).

To validate the results of RNA-Seq, 13 DEGs (3 upregulated and 10 downregulated genes) were randomly selected and assayed by qRT-PCR. In Figure 2C, 12 DEGs showed the same trend in both RNA-Seq analysis and qRT-PCR; the coincidence rate between qRT-PCR and RNA-Seq data was 92.31%, suggesting that transcriptome analysis was accurate and reliable.

### 2.4. Functional Classification of DEGs between SXCMS5A and SXCMS5B

Through analysis of gene ontology (GO) function, with Q value ≤ 0.05 as the threshold, 324 DEGs were annotated to 479 GO terms in the biological process, 28 of which were significantly enriched, and the first 5 GO terms were external encapsulation structure organization, cell wall organization, cell wall organization or biogenesis, carbohydrate metabolic process and cell wall modification (Table S3). A total of 418 DEGs were annotated to 335 GO terms in molecular functions, 25 of which were significantly enriched. The first 5 GO terms were enzyme inhibitor activity, molecular function regulator, enzyme regulator activity, catalytic activity and pectinesterase activity (Table S4). In addition, 131 DEGs were annotated to 111 GO terms in the cell components, 7 of which were significantly enriched, and the first 5 GO terms were cell wall, external encapsulation structure, membrane, cell peripheral, and intrinsic component of membrane (Table S5).

**Figure 3:**
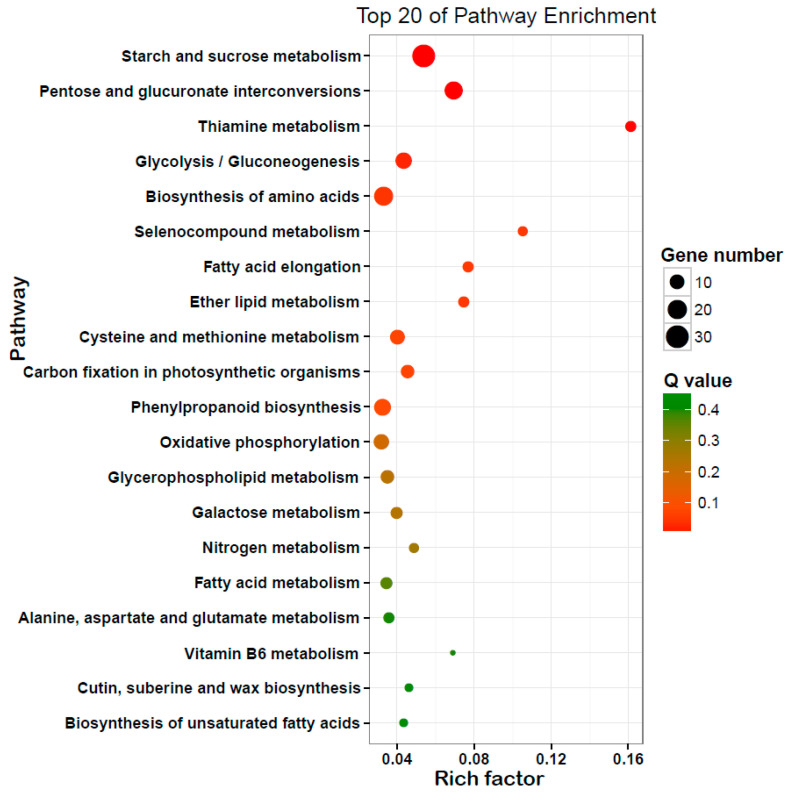
Top 20 Kyoto encyclopedia of genes and genome (KEGG) pathway analysis of DEGs between SXCMS5A and SXCMS5B. The *x*-axis indicated the rich factor corresponding to each pathway and the *y*-axis indicated name of the KEGG pathway. The dot color represented the Q values of the enrichment analysis. The size and color of bubbles represented the number and degree of enrichment of DEGs, respectively.

To identify the metabolic pathways in which the DEGs were involved and enriched, pathway analysis was performed using the Kyoto encyclopedia of genes and genomes (KEGG) pathway database. The results showed that starch and sucrose metabolism, pentose and glucuronate interconversions, thiamine metabolism, glycolysis/gluconeogenesis, biosynthesis of amino acids, and selenocompound metabolism were the main metabolic pathways (Figure 3).

### 2.5. Identification of DEGs Associated with Mitochondrial Genome

**Figure 4:**
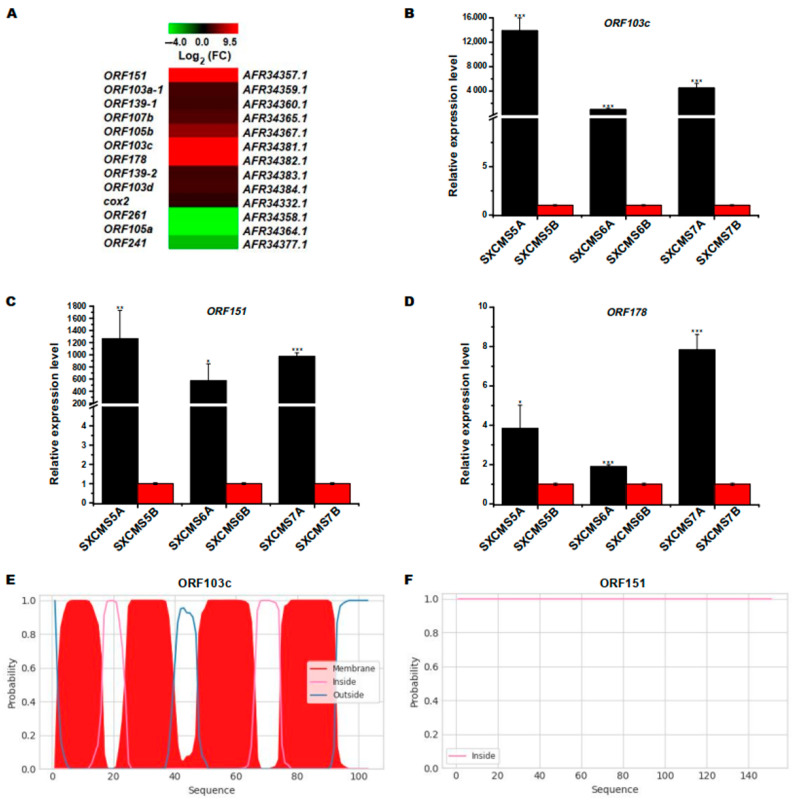
Analysis of DEGs in mitochondrial genome between soybean CMS lines and their maintainer lines. (**A**) Heat map of DEGs in mitochondrial genome between SXCMS5A and SXCMS5B. The heat map was conducted using MeV 4.9 software. Log_2_(FC) values were obtained from the RNA-seq data. (**B**–**D**) Relative expression level of *ORF103c*, *ORF151* and *ORF178* between soybean CMS lines and their maintainer lines. by qRT-PCR analysis. Asterisk indicated statistical significance: *, *p* < 0.05; **, *p* < 0.01; ***, *p* < 0.001. (**E**,**F**) Transmembrane domain analysis of ORF103c and ORF151. The abscissa indicated the amino acid length of ORFs. The ordinate represented the probability of the predicted transmembrane domain.

Numerous open reading frames (ORFs) in the mitochondrial genome were closely correlated with plant CMS [14]. In this study, 13 DEGs, i.e., 12 ORFs and 1 *COX2* in the soybean mitochondrial genome were differentially expressed between SXCMS5A and SXCMS5B (Table S2; Figure 4A), 10 out of the 13 genes were upregulated in SXCMS5A compared to SXCMS5B. Interestingly, 3 ORFs (*ORF151*, *ORF103c* and *ORF178*) were expressed at very low levels or not expressed in SXCMS5B. Especially, qRT-PCR analysis confirmed that these 3 ORFs genes were upregulated in SXCMS5A, SXCMS6A and SXCMS7A (the latter two CMS lines having a same cytoplasm source as SXCMS5A) compared to their respective maintainer SXCMS5B, SXCMS6B and SXCMS7B (Figure 4B–D). Furthermore, He et al. [22] found that *ORF178* was formed during the process of genome recombination in a soybean CMS line NJCMS1A. This indicated that *ORF178* might be a CMS gene of soybean. Since ORF103c also contains a transmembrane domain like ORF178 (Figure 4E,F and Figure S2) [22], *ORF103c* might also be a CMS gene of soybean.

### 2.6. Identification of DEGs Associated with Pollen Development

**Figure 5:**
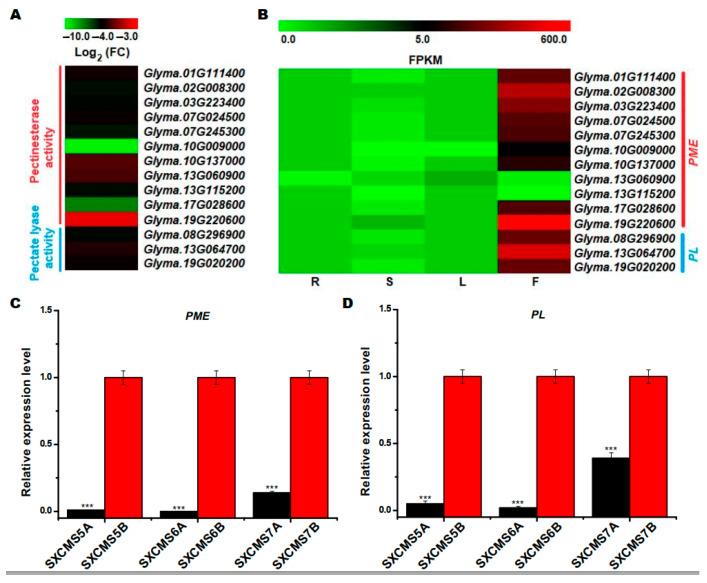
Analysis of DEGs related to pollen wall development between soybean CMS lines and their maintainer lines. (**A**) Heat map of the DEGs related to pollen wall development between SXCMS5A and SXCMS5B. The heat map was created using MeV 4.9 software. Log_2_(FC) values were obtained from RNA-seq data in this study. (**B**) Heat map of the DEGs related to pollen wall development in four different tissues. The color scale represented the relative transcript abundance of the DEGs in four soybean tissues. The heat map was created using MeV 4.9 software. Fragments per kilobase of transcript per million mapped reads (FPKM) values were obtained from RNA-seq data in Phytozome v12.0. R, root; S, stem; L, leaf; F, flower. (**C**,**D**) Relative expression level of *GmPME* (*Glyma.02G008300*) and *GmPL* (*Glyma.13G064700*) between soybean CMS lines and their maintainer lines by qRT-PCR analysis. PME, Pectin methylesterase; PL, pectate lyase. Asterisk indicated statistical significance: ***, *p* < 0.001.

Pectin methylesterase (PME, also named pectinesterase) and pectate lyase (PL) were two key enzymes involved in the degradation of plant pectin, and played important roles in the regulation of pollen development [23,24]. As noted above, 11 DEGs and 3 DEGs were found associated with pectinesterase activity (GO:0030599) and pectate lyase activity (GO:0030570) GO terms, respectively (Table S4). As shown in Figure 5A, all the 14 DEGs were downregulated in SXCMS5A compared to SXCMS5B. Most importantly, RNA-seq data in Phytozome v12.0 indicated all these transcripts were enriched in soybean flowers (Figure 5B). *GmPME* (*Glyma.02G008300*) and *GmPL* (*Glyma.13G064700*) were selected for qRT-PCR analysis, which were all downregulated in SXCMS5A, SXCMS6A and SXCMS7A compared to their respective maintainer SXCMS5B, SXCMS6B and SXCMS7B (Figure 5C,D). These findings suggested that the two gene types might involve in pollen development processes and that their reduced expression in soybean CMS might lead to pollen abortion.

### 2.7. Identification of DEGs Associated with Carbohydrate Metabolism and Sugar Transport

**Figure 6:**
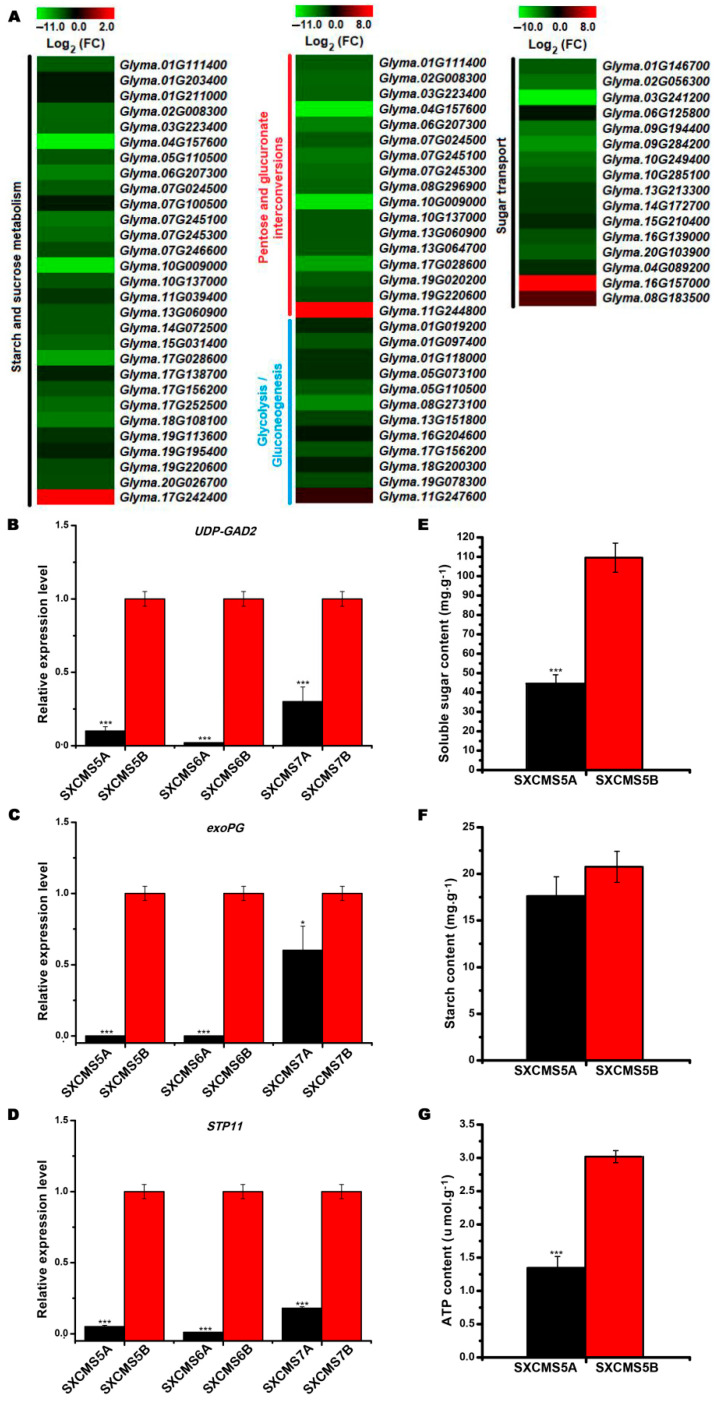
Analysis of DEGs related to carbohydrate metabolism and sugar transport between soybean CMS lines and their maintainers. (**A**) Heat map of the DEGs related to starch and sucrose metabolism, pentose and glucuronate interconversions, glycolysis/gluconeogenesis, and sugar transport between SXCMS5A and SXCMS5B. The heat map was created using MeV 4.9 software. Log_2_(FC) values were obtained from RNA-seq data. (**B**–**D**) Relative expression level of *UDP-glucuronic acid decarboxylase 2-like* (*UDP-GAD2*, *Glyma.07G246600*), *exopolygalacturonase* (*exoPG*, *Glyma.07G245100*) and *sugar transport protein 11* (*STP11*, *Glyma.20G103900*) between soybean CMS lines and their maintainers by qRT-PCR analysis. (**E**–**G**) Soluble sugar, starch, and adenosine triphosphate (ATP) contents analysis between SXCMS5A and SXCMS5B. Asterisk indicated statistical significance: *, *p* < 0.05; ***, *p* < 0.001.

Many DEGs between SXCMS5A and SXCMS5B involved in carbohydrate metabolism during flower bud development. Among these DEGs, 29, 17, and 12 were associated with starch and sucrose metabolism, pentose and glucuronate interconversions, and glycolysis/gluconeogenesis pathways, respectively (Figure 6A). Especially, most of these genes (55/58) were downregulated in SXCMS5A compared to SXCMS5B. *UDP-glucuronic acid decarboxylase 2-like* (*UDP-GAD2*, *Glyma.07G246600*), *exopolygalacturonase* (*exoPG*, *Glyma.07G245100*) were selected for qRT-PCR analysis, which were all downregulated in SXCMS5A, SXCMS6A and SXCMS7A compared to their respective maintainer SXCMS5B, SXCMS6B and SXCMS7B (Figure 6B,C). In addition, 16 DEGs were involved in sugar transport, and 14 of these were downregulated in SXCMS5A compared to SXCMS5B (Figure 6A). *Sugar transport protein 11* (*STP11*, *Glyma.20G103900*) was selected for qRT-PCR analysis, which was downregulated in SXCMS5A, SXCMS6A, and SXCMS7A, compared to their respective maintainer SXCMS5B, SXCMS6B, and SXCMS7B (Figure 6D). Furthermore, we measured soluble sugar, starch, and adenosine triphosphate (ATP) amounts in flower buds of SXCMS5A and SXCMS5B. The results showed that soluble sugar and ATP amounts decreased in SXCMS5A, while the starch amount decreased slightly in SXCMS5A, relative to SXCMS5B (Figure 6E–G). All these findings suggested that inhibition of carbohydrate metabolism and sugar transport might be two of the causes of soybean CMS.

### 2.8. Identification of DEGs Associated with Reactive Oxygen Species (ROS) Metabolism

**Figure 7:**
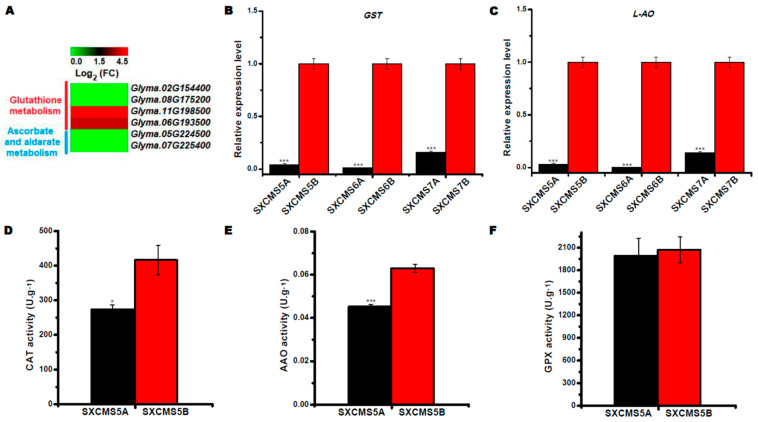
Analysis of DEGs related to reactive oxygen species (ROS) metabolism between soybean CMS lines and their maintainers. (**A**) Heat map of the DEGs related to glutathione metabolism and ascorbate and aldarate metabolism between SXCMS5A and SXCMS5B. The heat map was created using MeV 4.9 software. Log_2_(FC) values were obtained from RNA-seq data. (**B**,**C**) Relative expression level of *Glutathione S-transferase-like* (*GST*, *Glyma.02G154400*) and *L-ascorbate oxidase homolog* (*L-AO*, *Glyma.07G225400*) between soybean CMS lines and their maintainers by qRT-PCR analysis. (**D**–**F**) Activity assays of catalase (CAT), ascorbic acid oxidase (AAO) and glutathione peroxidase (GPX) between SXCMS5A and SXCMS5B. Asterisk indicated statistical significance: *, *p* < 0.05; ***, *p* < 0.001.

Several DEGs were found involved in ROS metabolism, including glutathione metabolism and ascorbate and aldarate metabolism (Figure 7A). Among these DEGs, 4 DEGs were associated with glutathione metabolism, and exactly half number of these genes were downregulated or upregulated in SXCMS5A compared to SXCMS5B. In addition, 2 DEGs were found associated with ascorbate and aldarate metabolism, and the RNA-seq showed that they were downregulated in SXCMS5A compared to SXCMS5B. *Glutathione S-transferase-like* (*GST*, *Glyma.02G154400*) and *L-ascorbate oxidase homolog* (*L-AO*, *Glyma.07G225400*) were selected for qRT-PCR analysis, which were downregulated in SXCMS5A, SXCMS6A, and SXCMS7A compared to their respective maintainer SXCMS5B, SXCMS6B, and SXCMS7B (Figure 7B,C). In addition, we measured catalase (CAT), ascorbic acid oxidase (AAO), and glutathione peroxidase (GPX) activities in flower buds of SXCMS5A and SXCMS5B. The results showed that CAT and AAO activities decreased in SXCMS5A, while the GPX activity was slightly decreased in SXCMS5A, relative to SXCMS5B (Figure 7D–F). All these findings suggested that downregulation of genes associated with ROS metabolism might be one of the causes of soybean CMS.

### 2.9. Identification of DEGs Associated with Transcription Factor

**Figure 8:**
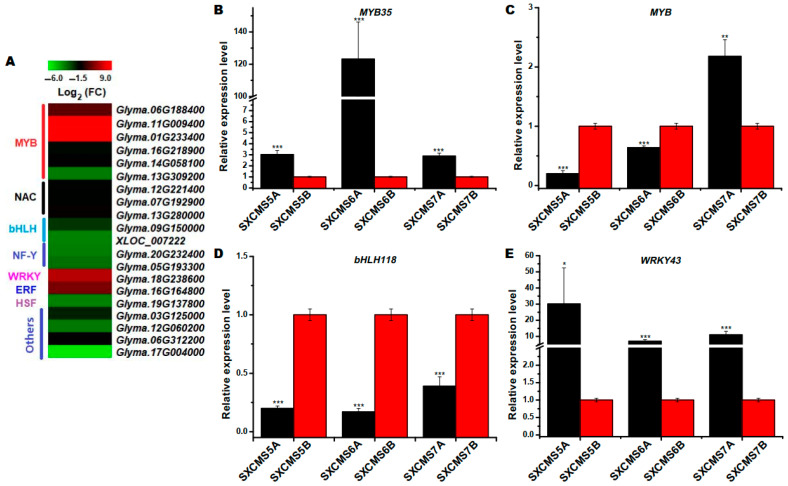
Analysis of DEGs related to transcription factors (TFs) between soybean CMS lines and their maintainers. (**A**) Heat map of the DEGs related to TFs between SXCMS5A and SXCMS5B. The heat map was created using MeV 4.9 software. Log_2_(FC) values were obtained from RNA-seq data. (**B**–**E**) Relative expression level of *GmMYB35* (*Glyma.06G188400*), *GmMYB* (*Glyma.16G218900*), *GmbHLH118* (*Glyma.09G150000*) and *GmWRKY43* (*Glyma.18G238600*) between soybean CMS lines and their maintainers by qRT-PCR analysis. Asterisk indicated statistical significance: *, *p* < 0.05; **, *p* < 0.01; ***, *p* < 0.001.

Many transcription factors (TFs) were differentially expressed between SXCMS5A and SXCMS5B. As shown in Figure 8A, a total of 20 differentially expressed transcription factors were found, including 15 downregulated and 5 upregulated TFs in SXCMS5A compared to SXCMS5B. The 15 downregulated DEGs were 3 MYB family TFs, 3 NAC family TFs, 2 bHLH family TFs, 2 nuclear family transcription factor Y subunit, 1 heat stress transcription factor B-3-like isoform X2, and 4 other TFs. The 5 upregulated DEGs were 3 MYB family TFs, 1 WRKY TF and 1 ethylene-responsive TF. Furthermore, *GmMYB35* (*Glyma.06G188400*), *GmMYB* (*Glyma.16G218900*), *GmbHLH118* (*Glyma.09G150000*) and *GmWRKY43* (*Glyma.18G238600*) were selected for qRT-PCR analysis. Among them, the expression trends of *GmMYB35* (*Glyma.06G188400*), *GmbHLH118* (*Glyma.09G150000*) and *GmWRKY43* (*Glyma.18G238600*) were consistent in SXCMS5A, SXCMS6A and SXCMS7A, compared to their respective maintainer SXCMS5B, SXCMS6B, and SXCMS7B (Figure 8B,D,E). However, the expression trend of *GmMYB* (*Glyma.16G218900*) was not consistent in SXCMS5A, SXCMS6A, and SXCMS7A compared to their respective maintainer SXCMS5B, SXCMS6B, and SXCMS7B (Figure 8C). This inconsistency suggested that *GmMYB* (*Glyma.16G218900*) might play different roles in different CMS-maintainer pairs. All these findings suggested that these TFs might involve in regulation of pollen development in soybean CMS.

## 3. Discussion

In plants, CMS, i.e., cytoplasmic nuclear interaction male sterility, is controlled by cytoplasmic and nuclear male sterility genes in a coordinated manner. It is generally believed that CMS is caused by the coupling of mitochondrial genes and nuclear genes [14]. The mitochondrial genome and nuclear genome locate on different parts in a cell, but they relate and influence each other in functions [25]. The nuclear genes encode various protein factors and enzymes needed for mitochondrial gene replication, transcription and translation, while mitochondrial gene mutation leads to the abnormality of its coding protein polypeptide, which can reverse-regulate the replication and expression of a series of nuclear genes through the signal pathway, leading to pollen abortion. In general, nuclear genes related to pollen wall development, carbohydrate metabolism and sugar transport, ROS metabolism and TFs play important roles in male fertility regulation. The relationship between DEGs and pollen abortion of SXCMS5A were discussed as follows.

### 3.1. ORF178 and ORF103c Identified as CMS Candidate Mitochondrial Genes of SXCMS5A

In plants, the production of most CMS was closely related to the variation, recombination and rearrangement of mitochondrial genome, resulting in a large number of new chimeric ORFs, which changed the transcription and translation products of genes, affected the expression and loss of function of genes, and led to male sterility in plants [12,26,27]. In rice, the CMS of the wild abortion type and Honglian type had been intensively investigated. Among the genes involved in these CMS variants, *WA352*, a CMS sterility gene, interacted with mitochondrial protein COX11 to stimulate the degradation of tapetum in anther, which in turn led to pollen abortion [28]. ORFH79, a protein expressed by a CMS sterility gene of the rice Honglian type, interacted with p61, a small subunit of mitochondrial respiratory electron transport chain complex III, resulting in ATP concentration decreased and ROS amount increased, which eventually led to cytoplasmic male sterility [29]. In this study, 13 DEGs included 12 ORFs and 1 *COX2* were mitochondrial genome genes between SXCMS5A and SXCMS5B of which three (*ORF103c*, *ORF151* and *ORF178*) were expressed almost exclusively in differential soybean CMS lines. Furthermore, both *ORF103c* and *ORF178* encode transmembrane proteins, which is one of the main characteristics of CMS genes [14,22]. Interestingly, *ORF178* was formed during the process of genome recombination in a soybean CMS line NJCMS1A, and it was also found that *ORF178* was expressed in NJCMS1A, NJCMS4A and NJCMS5A [22]. However, *ORF103c* was found in SXCMS5A, SXCMS6A and SXCMS7A, but not in NJCMS1A. In addition, *ORF261*, another CMS candidate gene in NJCMS1A, was downregulated in SXCMS5A compared to SXCMS5B, which might be caused by cytoplasmic differences. Thus, the upregulated expression of *ORF178* and *ORF103c* might change the transcription and translation products of genes and affect gene expression and loss of function, which was related to pollen abortion and male sterility in SXCMS5A. Since the role of these DEGs in pollen development had not been previously reported, these findings offered a new direction for investigations of the molecular mechanisms underlying soybean CMS.

### 3.2. Down-Regulation of DEGs Associated with Pollen Wall Development Is One of the Key Factors of Pollen Development Defect in SXCMS5A

The development of the pollen wall in pollen grains was a requirement for plant sexual reproduction, and most of the characters associated with male sterility were related to abnormal development of the pollen wall [30]. Pectin metabolism played an important role in pollen development; thus, inhibition of pectin metabolism during pollen development would lead to delayed pollen development, male sterility, and a lower seed setting rate [31,32]. In this study, we identified 11 PMEs, all of which were downregulated in the CMS line SXCMS5A compared to SXCMS5B. These genes were predicted to be correlated with pectinesterase activity. In addition, three PLs were also downregulated in the CMS line SXCMS5A compared to SXCMS5B. PL and PME were two important enzymes involved in the degradation of plant pectin and the formation of pollen walls in plants [23,24]. PL, Exo-PG, and PME were associated with male fertility restoration of the CMS line in pepper, and PL and PME played an important role in pollen development [33]. Thus, the downregulated expression of PL and PME genes might result in abnormal pollen wall development, which was related to pollen abortion and male sterility in SXCMS5A.

### 3.3. Blocked Carbohydrate Metabolism and Sugar Transport Leads to Abnormal Pollen Development in SXCMS5A

Carbohydrate metabolism and sugar transport were the most basic metabolic processes in plant, providing energy and carbon for anther development, and starch and sucrose serve as energy reserves for pollen maturation [34,35]. In this study, starch and sucrose metabolism, pentose and glucuronate interconversions, and glycolysis/gluconeogenesis were three enriched pathways for carbohydrate metabolism. These 3 key pathways contained 58 DEGs of which 55 were downregulated in SXCMS5A. Male sterility in the soybean CMS line NJCMS1A was associated with these three pathways [21]. Similarly, we found that many sugar transport-related DEGs, such as *STP11* and *sucrose transport protein SUC4-like* (*SUC4*), were downregulated in SXCMS5A. In cucumber, Sun et al. [16] found that downregulation of the sugar transporter *CsSUT1* inhibited pollen germination and caused male sterility. In soybean CMS-based F_1_, Ding et al. [36] had found downregulation of sugar transport-related DEGs and reduction of sugar accompanied by the decrease of male fertility under heat stress. Furthermore, substance amount analysis also showed that energy supply was decreased in SXCMS5A compared to SXCMS5B. Thus, the downregulated expression of carbohydrate metabolism and sugar transport related genes might lead to insufficient energy supply, which was related to pollen abortion and male sterility in SXCMS5A.

### 3.4. Abnormal ROS Metabolism Leads to Pollen Abortion in SXCMS5A

PCD was a common phenomenon in the development of animals and plants, regulated by genes under specific physiological or pathological conditions [37]. Tapetum provides nutrients for pollen development, and its abnormal PCD process is one of the direct causes of plant male sterility [14,15,38,39]. In this study, cytological analysis showed that the tapetum was vacuolated and abnormally developed in SXCMS5A compared to SXCMS5B, which had typical morphological characteristics of abnormal PCD. Although the male-sterile lines formed microspores through meiosis, they could not provide related materials for the development of pollen and could not ultimately form functional pollen. Most importantly, there was a close relationship between PCD and ROS metabolism [40,41]. Studies had shown that abnormal ROS metabolism during anther or spikelet development was related to male sterility [42,43,44]. Ascorbic acid and glutathione had important physiological functions in plants, with special roles in maintaining the redox balance of cells in the plant antioxidant system [45,46]. In this study, *L-AO* and *GST* genes (components of ascorbic acid and glutathione metabolism, respectively) were downregulated in flower buds of the soybean CMS lines compared to their maintainer lines. Furthermore, enzyme activity analysis also showed that ROS scavenging were decreased in SXCMS5A compared to SXCMS5B. Thus, the downregulated expression of ROS metabolism genes might lead to abnormal PCD, which was related to pollen abortion and male sterility in SXCMS5A.

### 3.5. Abnormal Expression of TF Related DEGs Causes Pollen Abortion in SXCMS5A

TFs are important regulators of gene expression, and their expression changes may have an important impact on plant growth and development [47,48]. For example, MYB, bHLH and WRKY participated in regulation of rates of gene transcription and regulation of meiosis, which was very important for stamen development and maturation [49,50,51,52]. Most of these TFs played a key role in the process of tapetum PCD and pollen formation, and their abnormal functioning often caused male sterility [13,53,54]. In this study, 20 coding TF DEGs were found between SXCMS5A and SXCMS5B among which 15 downregulated and 5 upregulated in SXCMS5A compared to SXCMS5B. Among these DEGs, 6 were related to MYB TFs, 2 were related to bHLH TFs, and 1 was related to WRKY TFs. In addition, effective activation of the ethylene signaling pathway was required for plant responses to growth and environmental signals, but continuous over activation of the ethylene signaling pathway had obvious inhibitory and toxic effects on plant growth and reproduction [55]. Previous research had shown that increasing ethylene concentration could lead to male sterility in wheat [55]. Up-regulation of the *Glyma.16G164800* encoding ethylene response transcription factor was also consistent with this result. Thus, the abnormal expression of these TFs might cause disturbance in expression of genes related to tapetum and pollen development, which was related to pollen abortion and male sterility in SXCMS5A.

### 3.6. Proposed Model for the Mechanism of Male Sterility in Soybean CMS Line SXCMS5A

**Figure 9:**
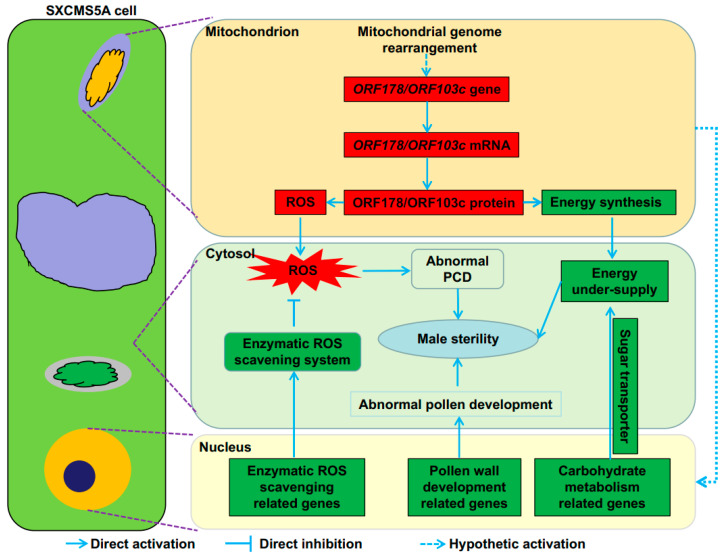
A proposed model for the mechanism of male sterility in soybean CMS line SXCMS5A. The upregulated and downregulated genes or metabolite contents are in red and green backgrounds, respectively.

According to previous reports and the data presented in this study, we made the following speculation on the mechanism of male sterility in soybean CMS line SXCMS5A (Figure 9). First, the rearrangement of soybean CMS line SXCMS5A mitochondrial genome generated CMS genes, including *ORF178* and *ORF103c*. The production of *ORF178* or *ORF103c* leads to mitochondrial dysfunction, such as blocked energy synthesis and massive production of ROS. Subsequently, mitochondrial defects directly/indirectly lead to the down-regulation of genes related to carbohydrate metabolism, sugar transport and pollen wall development in the nucleus, leading to further energy shortage and abnormal pollen development during pollen development. In addition, the downregulation of enzymatic ROS scavenging related genes in the nucleus leads to the dysfunction of the enzymatic ROS scavenging system, resulting in the inability of effective ROS clearance and the accumulation of ROS, which affects the normal PCD process of anther tapetum. The combination of these processes eventually leads to male sterility in soybean CMS line SXCMS5A. Further studies are needed to validate this proposed model.

## 4. Materials and Methods

### 4.1. Plant Materials and Sample Collection

Three soybean CMS lines, SXCMS5A, SXCMS6A, and SXCMS7A with their respective maintainer lines were used in the present study. SXCMS5A was developed by continuous backcross with the CMS line H3A as the donor parent and the variety JY20 (designated as SXCMS5B afterwards) as the recurrent parent; SXCMS6A was developed by continuous backcross with the CMS line H3A as the donor parent and the strain LX11 (designated as SXCMS6B afterwards) as the recurrent parent; and SXCMS7A was developed by continuous backcross with the CMS line H3A as the donor parent and the strain JDX (designated as SXCMS7B afterwards) as the recurrent parent. Here, the CMS line H3A was developed by continuous backcross with the CMS line JLCMS1A as the donor parent and the strain H3 as the recurrent parent, whereas JLCMS1A was introduced from Jilin Academy of Agricultural Sciences.

SXCMS5A and SXCMS5B were planted in the summer of 2017 at Dangtu Experimental Station of Nanjing Agricultural University. Because it was difficult to judge the precise pollen development stage from flower bud appearance in soybean, the mixture of flower buds with different sizes were collected from three individual plants in the afternoon as three biological replicates for SXCMS5A and SXCMS5B. The samples were immediately placed in liquid nitrogen and then stored at −80 °C for RNA-Seq.

SXCMS5A and SXCMS5B, SXCMS6A and SXCMS6B, and SXCMS7A and SXCMS7B were planted in the summer of 2019 at Dangtu Experimental Station. The mixture of flower buds with different sizes were collected in the afternoon during flowering period, immediately placed in liquid nitrogen, then stored at −80 °C for qRT-PCR. All qRT-PCR reactions were performed with three biological replicates.

SXCMS5A and SXCMS5B were planted in the spring of 2019 at Dongyang Experimental Station of Shanxi Agricultural University. Flower buds with different sizes were collected in the afternoon at the flowering stage and fixed in formaldehyte-alcohol-acetic acid (FAA) for cytological examination. The mixture of flower buds of different sizes was collected in the afternoon at the flowering stage, immediately placed in liquid nitrogen, then stored in at −80 °C for enzyme activity assay and substance content analysis. All enzyme activity assay and substance content analysis were performed with three biological replicates.

### 4.2. Cytological Examination

To observe the cyto-morphological characteristics of pollen development of SXCMS5A and SXCMS5B, flower buds with different sizes were fixed, dehydrated, embedded, sectioned and stained according to a previous report [56]. To observe the pollen fertility of SXCMS5A and SXCMS5B, the anthers of unopened flowers (the flowers that will open in the morning of next day) in the afternoon were taken and stained with a 1% I_2_-KI solution [57]. All samples were observed using a light microscope (Nikon Eclipse CI, Tokyo, Japan), and photographed under the imaging system (Nikon DS-U3, Tokyo, Japan).

### 4.3. Total RNA Extraction, Library Construction, and Sequencing

Trizol (Invitrogen, Carlsbad, CA, USA) was used to extract total RNA from the flower buds of SXCMS5A and SXCMS5B. The construction of cDNA library referred to prokaryote, considering that the plant mitochondrial genomes were similar to its ring genome. So, after total RNA was extracted, sample mRNA was enriched by removing rRNA by a Ribo-ZeroTM Magnetic Kit (Epicenter, Madison, WI, USA). Next, the enriched mRNA was fragmented into short fragments using fragmentation buffer and reverse-transcribed into cDNA with random primers. Second-strand cDNA was synthesized with DNA polymerase I, RNase H, dNTPs, and buffer. The cDNA fragments were then purified with a QiaQuick PCR extraction kit, end-repaired, poly(A) was added, and ligated to Illumina sequencing adapters. The ligation products were size selected by agarose gel electrophoresis, PCR amplified, and sequenced using Illumina HiSeqTM 2500 by Gene Denovo Biotechnology Co. (Guangzhou, China).

### 4.4. Raw Sequencing Data Analysis and Bioinformatics Analysis

The raw data from the sequencing machines were initially filtered to get clean data. The short-reads alignment tool Bowtie2 [58] was used to compare and remove reads containing rRNA. Tophat2 software [59] was used to compare the reads of the filtered rRNA to the reference genome (Nucleus, Wm82.a2.v1; mitochondria, JX463295.1; chloroplast, DQ317523.1). Next, the transcripts of a group of different repeats were fused into comprehensive transcripts with Cufflinks software [60]; transcripts of multiple groups were then merged into a group of final comprehensive transcripts for further analysis of downstream differential expression. Transmembrane domain of ORF was predicted using DeepTMHMM (V.1.0.12, https://dtu.biolib.com/DeepTMHMM, accessed on 15 September 2022) [61]. The FPKM values of DEGs between SXCMS5A and SXCMS5B in soybean root, stem, leaf and flower tissues were obtained from the RNA-seq data in Phytozome v12.0 (https://phytozome.jgi.doe.gov/pz/portal.html#, accessed on 15 August 2020), and the heat map was conducted using MeV 4.9 software.

### 4.5. Quantification of Gene Abundance and DEG-Analysis

Gene abundance was quantified with RSEM software [62]. The gene expression level was normalized with fragments per kilobase of transcript per million mapped reads (FPKM) method. To identify differentially expressed genes, the edgeR package was used. A standard of “FDR < 0.05 and |log_2_FC| > 1” was used as the threshold to screen for significant DEGs. DEGs were then subjected to enrichment analysis of GO functions and KEGG pathways.

### 4.6. GO and KEGG Pathway Enrichment Analysis

GO enrichment analysis identified all GO terms that were significantly enriched in DEGs comparing to the genome background, and filtered the DEGs that correspond to biological functions. All DEGs were mapped to GO terms in the gene ontology database (http://www.geneontology.org/, accessed on 15 December 2017); gene numbers were calculated for every term, and significantly enriched GO terms in DEGs compared to the genome background were defined by hypergeometric test. The calculated *p* values were run through FDR correction, taking a Q value ≤ 0.05 as a threshold. GO terms meeting this condition were defined as significantly enriched GO terms in DEGs. This analysis enabled identification of the main biological functions correlated with the DEGs in question.

KEGG was the major public pathway-related database [63]. Pathway enrichment analysis identified significantly enriched metabolic pathways or signal transduction pathways in DEGs compared to the whole genome background. The calculated *p* value was run through FDR correction, taking a Q value ≤ 0.05 as a threshold value. Pathways meeting this condition were defined as significantly enriched pathways in DEGs.

### 4.7. qRT-PCR Analysis

qRT-PCR was used to validate the gene expression levels of DEGs detected by RNA-Seq. All primers (Table S6) were designed based on the mRNA sequences, and synthesized commercially (General Biosystems, Chuzhou, China). Total RNAs from the flower buds of SXCMS5A and SXCMS5B, SXCMS6A and SXCMS6B, SXCMS7A and SXCMS7B were used for the validation of RNA-Seq. Using the protocol provided in the HiScript Q RT SuperMix for qPCR kit (+gDNA wiper, Vazyme, Nanjing, China), 1 μg of total RNA was reverse-transcribed using oligo (dT) primers. qPCR analysis was carried out using the AceQ qPCR SYBR Green Master Mix (Vazyme, Nanjing, China) on a Bio-Rad CFX96 instrument (CFX96 Touch, BIO-RAD, USA). *GmTubulin* (accession number: *NM_001252709.2*) was used as the internal control gene [36]. The maintainer lines were used as the control of their male sterile lines. Relative expression levels of the genes were quantified using the 2^−ΔΔCt^ method [64].

### 4.8. Substance Contents and Enzyme Activity Assay

Soluble sugar and starch contents were measured by visible light spectrophotometer according to the operation procedure of soluble sugar content detection kit (Solarbio, Beijing, China) and starch content detection kit (Solarbio, Beijing, China), respectively. ATP content was measured by UV spectrophotometer according to the operation procedures of ATP content detection kit (Solarbio, Beijing, China).

CAT and AAO activities were measured by UV spectrophotometer according to the operation procedure of CAT activity test kit (Solarbio, Beijing, China) and AAO activity test kit (Solarbio, Beijing, China), respectively. GPX activity was measured by visible light spectrophotometer according to the operation procedure of GPX activity test kit (Solarbio, Beijing, China).

## 5. Conclusions

In this study, two ORFs in mitochondria, including *ORF178* and *ORF103c*, were upregulated in sterile lines and had transmembrane domains, which were identified as two candidate CMS genes of soybean CMS line SXCMS5A as well as its two half-sib sister lines with a same cytoplasm source (SXCMS6A and SXCMS7A). Our study showed that pollen wall development, carbohydrate metabolism, sugar transport, ROS metabolism related genes and TFs were involved in the process of pollen abortion and male sterility. The male sterility mechanism of SXCMS5A might be the rearrangement of soybean mitochondrial genome to produce CMS gene, which directly or indirectly affected a series of biological processes, such as the decrease of energy supply and the outbreak of ROS, leading to the abnormal development of anther tapetum and finally pollen abortion. Future research will focus on cloning CMS related candidate genes in soybean.

## Data Availability

The datasets generated by this study can be found in the NCBI using accession number PRJNA887481.

## Supplementary Materials

The following supporting information can be downloaded at: https://www.mdpi.com/article/10.3390/ijms232012227/s1.
