# Supplementary material for: Transcriptome Analysis Reveals the Genes Related to Pollen Abortion in a Cytoplasmic Male-Sterile Soybean (Glycine max (L.) Merr.)
Source: Int J Mol Sci. 2022 Oct 13;23(20):12227. doi: 10.3390/ijms232012227 (PMC9603564; doi:10.3390/ijms232012227)
Supplement: Supplementary file 1 [file ijms-23-12227-s001.zip › Supplementary files/Supplement Figures.pdf]

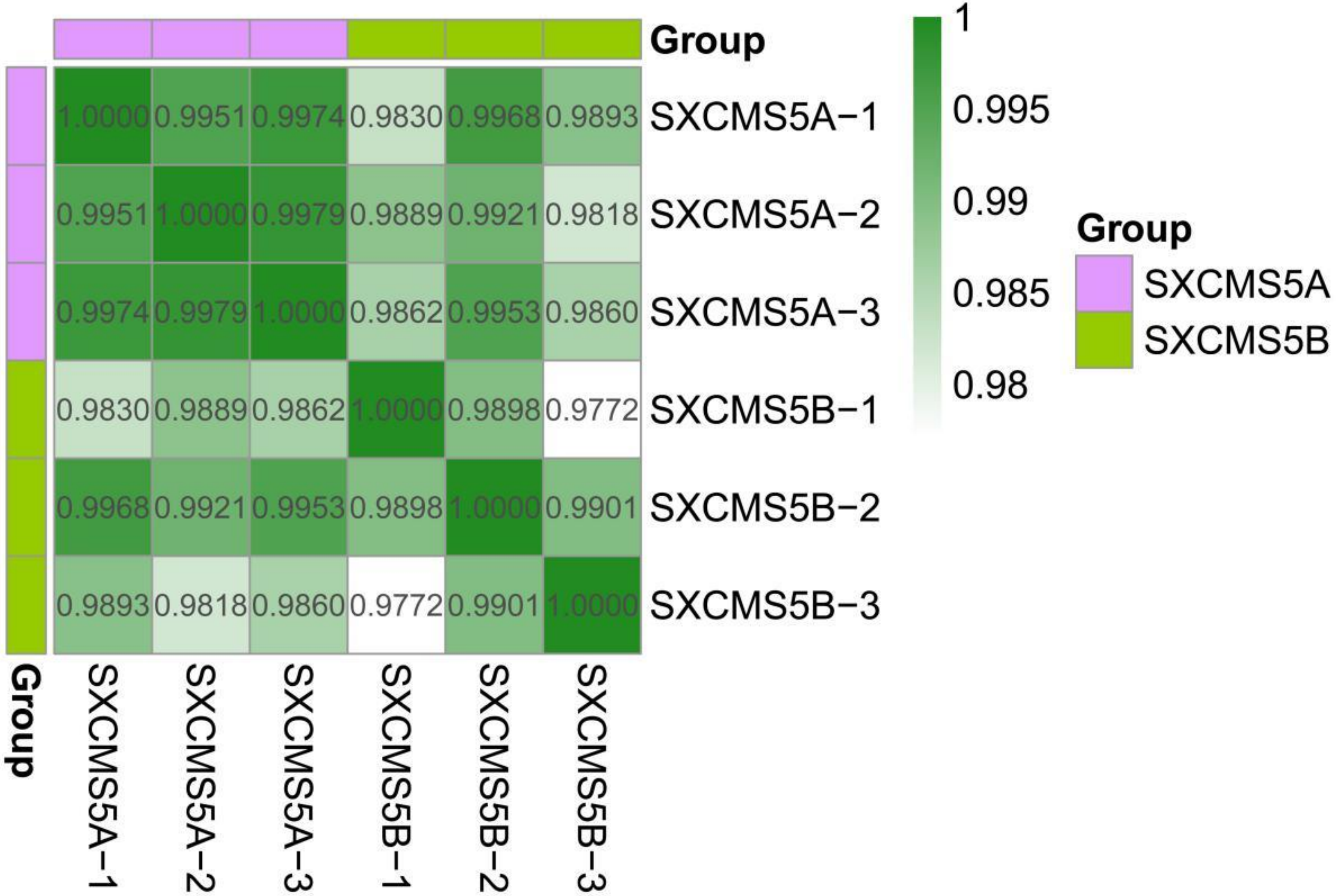

Figure S1. The repeated correlation assessment analysis of soybean CMS line SXCMS5A and its maintainer SXCMS5B

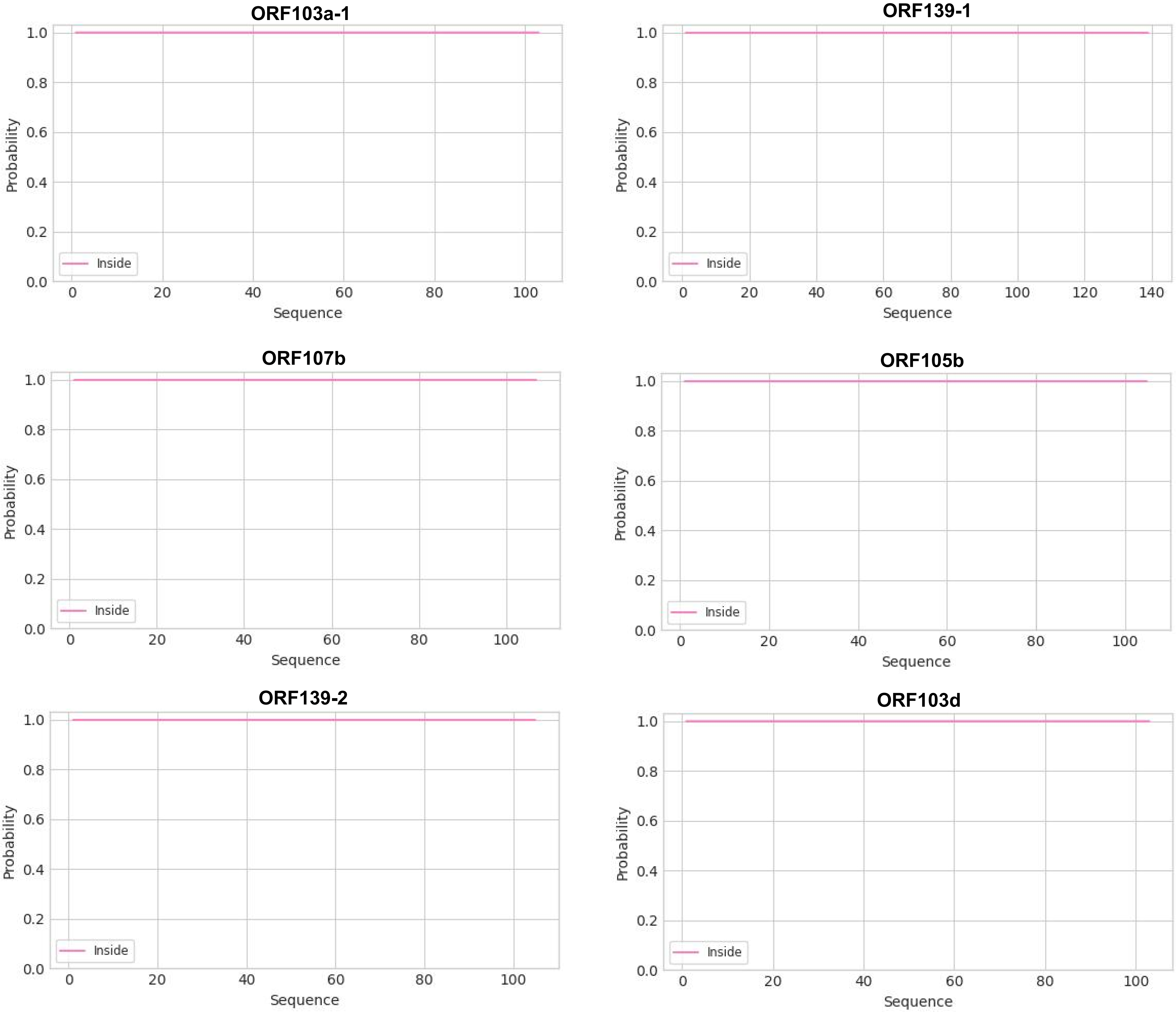

Figure S2. Transmembrane domain analysis of ORF103a-1, ORF139-1, ORF107b, ORF105b, ORF139-2 and ORF103d. The abscissa indicated the amino acid length of ORFs. The ordinate represented the probability of the predicted transmembrane domain.
